# Emergence and evolution of mosaic *penA-60* and *penA-237* alleles in a *Neisseria gonorrhoeae* core genogroup that was historically susceptible to extended spectrum cephalosporins

**DOI:** 10.3389/fmicb.2024.1401303

**Published:** 2024-10-01

**Authors:** Jesse C. Thomas IV, John C. Cartee, Katherine Hebrank, Sancta B. St. Cyr, Karen Schlanger, Brian H. Raphael, Ellen N. Kersh, Sandeep J. Joseph

**Affiliations:** ^1^Division of STD Prevention, Centers for Disease Control and Prevention, Atlanta, GA, United States; ^2^Oak Ridge Institute for Science and Education Research Participation and Fellowship Program, Oak Ridge, TN, United States; ^3^Division of HIV Prevention, Centers for Disease Control and Prevention, Atlanta, GA, United States

**Keywords:** *Neisseria gonorrhoeae* (Ng), antibiotic resistance, phylogenomics, ceftriaxone resistance, homologous recombination

## Abstract

**Introduction:**

*Neisseria gonorrhoeae* (Ng) has successively developed resistance to all previously recommended antimicrobial therapies, with ceftriaxone being the last option for monotherapy of gonorrhea. Global emergence and international spread of the FC428 clone derived mosaic *penA-60* allele, associated with highlevel ceftriaxone minimum inhibitory concentrations (MICs) in non FC428 clone Ng lineages, has become an increasing concern. The *penA-60* allele carrying Ng was first identified in the U.S. in Las Vegas, Nevada (2019; GCWGS-102723), with a multi-locus sequence type (MLST)-1901 strain, in a non FC428 clone Ng lineage, which is associated with a historically ceftriaxone susceptible core genogroup. Later in 2022, an allele genetically similar to *penA-60*, mosaic *penA-237*, was identified in the UK (H22-722) and France (F92) with high-level ceftriaxone MICs and both belonged to MLST-1901.

**Methods:**

In this study, we assessed phylogenomic relatedness and antimicrobial resistance (AMR) determinant profiles of these three isolates with high-level ceftriaxone MICs among a global collection of 2,104 genomes belonging to the MLST-1901 core genome cluster group 31, which includes strains separated by a locus threshold of 200 or fewer differences (Ng_cgc_200). Recombination events in and around the *penA* coding region were catalogued and potential sources of inter species recombinant DNA were also inferred.

**Results:**

The global population structure of MLST-1901 core genogroup falls into 4 major lineages. Isolates GCWGS-10723, F92, and H22-722 clustered within Lineage 1, which was dominated by non-mosaic penA-5 alleles. These three isolates formed a clade within Lineage 1 that consisted of isolates from North America and southeast Asia. *Neisseria subflava* and *Neisseria sicca* were identified as likely progenitors of two independent recombination events that may have led to the generation of mosaic *penA-60* and *penA-237*, within a possible non-mosaic *penA-5* background.

**Discussions:**

Our study suggests that there are multiple evolutionary pathways that could generate concerning mosaic *penA* alleles via homologous recombination of historically susceptible Ng lineages with *Neisseria* commensals. Enhanced surveillance of gonococcal strains and *Neisseria* commensals is crucial for understanding of the evolution of AMR, particularly in less-studied regions (e.g., Asia), where high-level ceftriaxone MICs and multi-drug resistance are more prevalent.

## Introduction

*Neisseria gonorrhoeae*, the etiological agent of the sexually transmitted infection (STI) gonorrhea, is the second most reported infectious disease in the United States (U.S.) (648,056 cases were reported to the CDC, 2024), with an estimated yearly incidence of 87 million infections worldwide (Rowley et al., 2019; CDC, 2024). *N. gonorrhoeae* antimicrobial resistance (AMR) has been classified as an “urgent threat” by the CDC due to the organism’s historic evolution of AMR to all previously recommended drug therapies used for treatment of gonorrhea (e.g., sulfonamides, penicillins, tetracyclines, macrolides, fluoroquinolones) (Centers for Disease Control and Prevention (U.S.), 2019). By the early-2000s, extended spectrum cephalosporins (ESCs) remained largely effective at low concentrations, however the 2010s saw the global emergence and spread of gonococcal strains with reduced susceptibility (RS) or minimum inhibitory concentration [MIC] values of greater than or equal to 0.25 μg/mL to oral cefixime (CFM) (Deguchi et al., 2003; Ito et al., 2004; Yokoi et al., 2007; Ohnishi et al., 2011; Cámara et al., 2012; Unemo et al., 2012; Unemo and Shafer, 2014; Shimuta et al., 2015). This left ceftriaxone (CRO), the only current cornerstone of therapy, as the only ESC recommended for first-line treatment of gonorrhea in the U.S. and other countries (Unemo and Shafer, 2014). Concerns over the future emergence of ceftriaxone-resistant gonorrhea, led several countries, including the U.S. in 2010, to recommend combination therapy with azithromycin (Workowski et al., 2015). In 2020, however, in response to increasing AMR to azithromycin, combination therapy was replaced in favor of ceftriaxone monotherapy in the U.S. (500 mg, 1 g for persons weighing ≥ 150 kg), as the only recommended regimen for uncomplicated gonorrhea (Cyr, 2020).

Decreased susceptibility to ESCs is associated with “mosaic” *penA* alleles, which contain 60 to 70 mutations in the second half of the gene, compared to wild-type genes (Ito et al., 2005). The novel ceftriaxone-resistant gonococcal strain H041 (mosaic *penA*-10) was first reported in Japan in 2009 (Ohnishi et al., 2011), which was followed by reports of ceftriaxone-resistant *N. gonorrhoeae* harboring other unique mosaic *penA* alleles from France (Unemo et al., 2012), Spain (Cámara et al., 2012) and Australia (Lahra et al., 2014). Although these strains did not spread further, the threat of ESC resistant *N. gonorrhoeae* was further complicated by the emergence and international spread of gonococcal strains with the FC428 derived mosaic *penA-60* allele first identified in Japan around 2015. This allele confers high-level cefixime (CFM^HL^;MICs of ≥ 1 μg/mL) and ceftriaxone MICs (CRO^HL^; MICs of ≥ 0.5 μg/mL) (Nakayama et al., 2016). FC428 clones have disseminated internationally and consist of closely related multi-locus sequence types (MLSTs), predominately MLST-1903, MLST-7365, MLST-1600, MLST-13871, MLST-13943 and MLST-13429, with reports from China [2015 to 2021; (Tang et al., 2023)], Australia [2017; (Lahra et al., 2018)], Canada [2017–2018; (Lefebvre et al., 2018; Berenger et al., 2019)], Denmark [2017; (Terkelsen et al., 2017)], Ireland [2018; (Golparian et al., 2018)], the UK [2018 (Eyre et al., 2019)], Singapore [2018; (Ko et al., 2019)] and France [2019; (Poncin et al., 2019)].

The mosaic *penA*-60 allele has also been identified in phylogenetically distinct gonococcal lineages. In 2018, three MLST-12039 isolates carrying the *penA-60* allele were reported in the UK [G97687 and G7944; (Eyre et al., 2018)] and Australia [A2735 and A2543; (Whiley et al., 2018)]. Since then mosaic *penA*-60 alleles have been identified in multiple non-FC428 lineages collected around the world, including the U.S. [MLST-1901; (Picker et al., 2020)], [MLST-8123; (Reimche et al., 2024)], Austria [MLST-16406; (Pleininger et al., 2022)], France [MLST-16406; (Maubaret et al., 2023)], the UK [MLSTs ST-16406, ST-8123 and ST-1901; (Day et al., 2022)], and Cambodia [MLSTs ST-8143, ST-16406 and ST-8123;(Ouk et al., 2023)]. Increasingly worrying is the emergence of mosaic *penA*-60 derivative alleles (sharing ≥ 98% sequence homology), such as mosaic *penA*-237 harbored by MLST-1901 strains, detected in France (Berçot et al., 2022) and the UK (Day et al., 2022), both with travel links to Southeast Asian countries.

In November 2019, the Southern Nevada Public Health Laboratory of the Southern Nevada Health District (SNHD) identified a male urethral gonococcal isolate (GCWGS-10723) with high-level MICs to cefixime (MIC = 2 μg/mL) and ceftriaxone (MIC = 1 μg/mL) (Picker et al., 2020). Molecular testing by the CDC revealed the presence of the mosaic *penA*-60 allele, which is the first case of its kind identified within the U.S. Interestingly, whereas the majority of mosaic *penA*-60 harboring strains have belonged to the clonally-expanding FC428 group (MLST ST1903 and similar strains), this particular isolate was revealed to be more closely related to the globally circulating MLST1901 lineage, commonly associated with resistance to ciprofloxacin and reduced susceptibility to azithromycin and cephalosporins (Osnes et al., 2021; Thomas et al., 2021). The main female partner of the patient reported travel to China and being treated for gonorrhea while in China (Picker et al., 2020). The emergence of this concerning isolate, in addition to the identification of two other phylogenetically related strains with similar travel history to Southeast Asia in 2022 (*penA*-237 harboring strains, F92 and H22-722), prompted us to perform an in-depth phylogenomic investigation in order to ascertain the factors that may have contributed to their emergence.

## Materials and methods

### Isolate selection and susceptibility testing

In total, 2,104 *N. gonorrhoeae* isolate genome sequences belonging to the MLST-1901 core genogroup cluster locus threshold of 200 or fewer differences (Ng_cgc_200, Group 31), available as of August 2023, were obtained from PubMLST.org (Jolley et al., 2018; Harrison et al., 2020). The isolates of concern (GCWGS-10723, F92 and H22-722) belonged to group 31. This core-genogroup includes the ST1901 strains and their closely related single or double locus MLST variants (∼53 different MLSTs), including many described in several previous studies (Demczuk et al., 2015; de Curraize et al., 2016; Costa-Lourenço et al., 2018; Lee et al., 2018; Ryan et al., 2018; Sánchez-Busó et al., 2019; Williamson et al., 2019; Yuan et al., 2019; Alfsnes et al., 2020; Banhart et al., 2021; Yahara et al., 2021). Of the 2,104 genomes, 997 were from Europe, 800 from North America, 145 from South America, 116 from Asia, 40 from Oceania, 1 from Africa and 5 unknown continent, spanning 28-years (1992-2022). This collection includes two isolates available in the World Health Organization (WHO) reference panel as WHO-Y (F89; France, 2010) and WHO-V (Sweden, 2012) (Unemo et al., 2016). Isolates from the U.S. (*n* = 688) were obtained primarily from the Gonococcal Isolate Surveillance Project (GISP), interspersed with smaller numbers obtained through Strengthening the US Response to Resistant Gonorrhea (SURRG) from 2002 to until 2019. All isolates in our global dataset, including data on antimicrobial susceptibility profiles, MLST, and associated AMR determinants are provided in Supplementary Table 1.

### Whole genome sequencing and bioinformatic analyses

All 688 *N. gonorrhoeae* isolates from GISP or SURRG were sequenced on an Illumina MiSeq or HiSeq sequencer at the CDC core sequencing facility (Atlanta, GA) or state public health laboratories, which are part of the Antibiotic Resistance (AR) Laboratory Network (i.e., Maryland, Texas, or Washington State regional laboratories). Quality assessment was performed using FastQC v0.10.1. All raw Illumina sequencing reads were initially screened using Kraken v0.10.5 (Wood and Salzberg, 2014) to identify reads assigned to bacterial strains other than *N. gonorrhoeae*. Samples with more than 10% of raw reads assigned to *Neisseria meningitidis* or other bacterial species were considered contaminated. CutAdapt v1.8.3 (Martin, 2011) was used to remove adapter sequences using a minimum quality threshold of < Q30 and a minimum length threshold of 19. Illumina reads were *de novo* assembled using Spades v3.15.0 (Bankevich et al., 2012) by passing the *–careful* option. All assemblies downloaded from PubMLST and other published sources were screened with Mash v2.2.2 (Ondov et al., 2016)^1^ for the presence of contigs from other bacterial taxa other than Ng, and their quality was assessed by generating basic assembly statistics such as number of contigs, N50, L50 and GC content using bbmap v38.87.^2^

AMR genotypes were determined using a combination of several tools, including a customized gonococcal-specific ARIBA v2.14.5^3^ database (Hunt et al., 2017) and using an in-house customized python script called the *Neisseria gonorrhoeae* Genome Profiler and Typing Tool (CDC) (Schmerer et al., 2020). InSilicoSeq v1.5.3^4^ was used to generate 1 million simulated reads per assembly, for samples for which no raw reads were available, based on a MiSeq error profile (Gourlé et al., 2019). MLST and NG-MAST sequence types (STs) were determined by MLST v2.19^5^ and NGMASTER v0.4,^6^ respectively, while NG-STAR v2.0 STs were determined using pyngSTar^7^ (Jolley and Maiden, 2010; Kwong et al., 2016; Demczuk et al., 2017). Sequence types based on the *N. gonorrhoeae* core-genome MLST (cgMLST) v1.0 scheme and the Ng_cgc_200 grouping scheme (Harrison et al., 2020) were obtained by querying or submitting assemblies to the PubMLST *Neisseria* database,^8^ which uses the Bacterial Isolate Genome Sequence Database (BIGsdb) software (Jolley et al., 2018).

**FIGURE 1:**
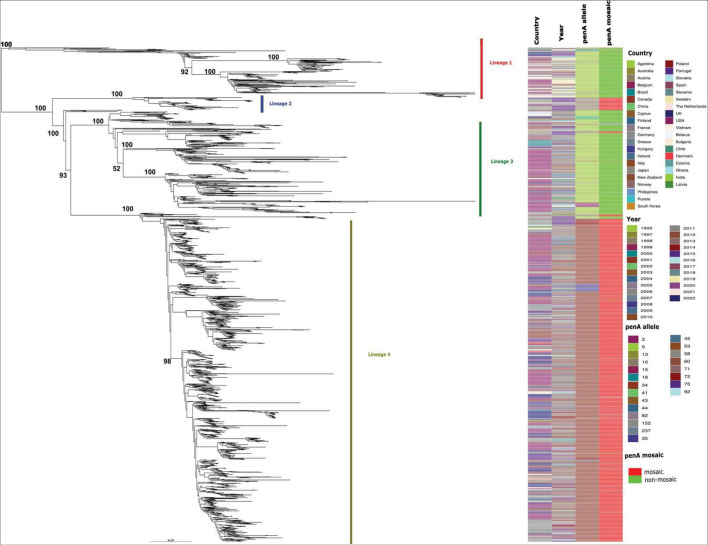
Maximum likelihood phylogenetic tree based on core genome SNPs of 2,104 MLST-1901-associated core-genome group 31 gonococci global isolates, sampled from 40 countries (including the US) between 1992 and 2022. All four major lineages are shown along with the country of origin, year of isolation, *penA* allele type, as well as whether the *penA* allele sequence were mosaic or non-mosaic. The bootstrap estimates (out of 100) for all the major internal nodes are also shown.

**FIGURE 2:**
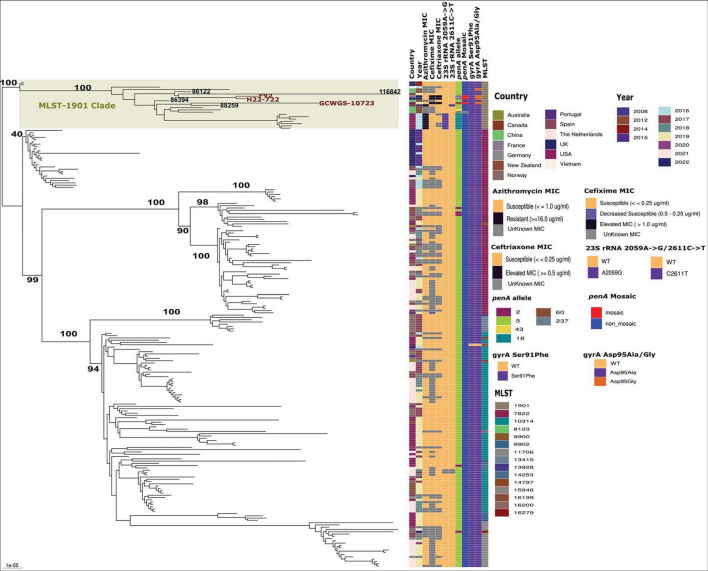
Maximum likelihood phylogenetic tree based on core genome SNPs of 213 MLST-1901-associated core-genome group 31 lineage 1 gonococci global isolates. Antimicrobial MICs, important AMR markers and MLSTs associated with each of the lineage 1 isolates are shown. The high-level ESC isolates carrying penA-60(GCWGS-10723) and penA-237(F92 and H22-722) within the MLST-1901 clade in lineage 1 are highlighted. The bootstrap estimates (out of 100) for all the major internal nodes are also shown.

For phylogenetic analysis, a core genome alignment was generated using Snippy v4.3.8^9^ with WHO-Y (GenBank accession number LT592161.1) provided as the reference (Unemo et al., 2016). This full-length whole genome alignment was used as an input to Gubbins v2.3.1 for identifying and filtering regions of homologous recombination (Croucher et al., 2014). The resulting alignment, containing only the polymorphisms present in the non-recombinant regions, was used as input for RAxML v8.2.9^10^ (Stamatakis, 2014), under the GTR+GAMMAX model of nucleotide substitution with a majority-rule consensus (MRE) convergence criterion, to reconstruct an ascertainment bias corrected (Stamatakis method) maximum-likelihood (ML) phylogeny. Two phylogenetic analyses were conducted, using the whole genome alignment generated from the 2104 *N. gonorrhoeae* isolates within the Ng_cgc_200 group 31 cluster (Figure 1), as well as a clade consisting of 213 *N. gonorrhoeae* isolates, where the isolates of interest (GCWGS-10723, F92 and H22-722) clustered (Figure 2), as described above. Pairwise SNP distances between isolate genomes were calculated with snp-dists v0.7,^11^ using the recombination masked alignment from Gubbins as input. All phylogenetic trees were visualized using the R packages ggtree v3.2.1 (Yu et al., 2017) and RCandy v1.0.0 (Chaguza et al., 2022).

Predicted homologous recombination events in and around the *murE* – *penA* coding regions for GCWGS-10723, F92 and H22-722 were visualized using RCandy (Chaguza et al., 2022). Essentially, Gubbins output files generated based on the whole genome alignment of the clade (n = 212), where the above mentioned three Ng isolates of concern clustered as described above, were imported into Rcandy and the unique and ancestral recombination events were visualized. fastGEAR (Mostowy et al., 2017) was also run with default parameters, to understand the flow of inter-species homologous recombination events that may have contributed to the emergence and acquisition of mosaic *penA-60* and *penA-237* alleles in GCWGS-10753 and in both F92 and H22-722 isolates, respectively. For fastGEAR analysis, a multi-species alignment of the *murE* – *penA* region for 14 *N. gonorrhoeae* MLST-1901 clade isolates, along with the homologous regions of *murE* – *penA* regions extracted from reference genomes using customized BLAST (Altschul et al., 1990) for the following commensal *Neisseria* species: *N. elongata* (NZ_CP031252.1), *N. flavescens* (NZ_CP039886.1), *N. subflava* (NZ_CP039887.1), *N. sicca* (NZ_CP072524.1), *N. mucosa* (NZ_UGRT01000006.1), *N. meningitidis* (CP018907.1; urethritis clade), *N. polysaccharea* (NZ_QQEZ01000004.1), *N. cinerea* (NZ_LS483369.1) *and N. lactamica* (NZ_CP031253.1), were generated using muscle v5.1.0^12^ (Edgar, 2004). FastGEAR infers the genetic population structure from a given alignment using a Hidden Markov Model. Populations are defined as groups which are genetically distinct in at least 50% of the alignment. Within each inferred population, recombination events are identified by comparing every nucleotide site in the target sequence to all remaining populations and asking whether it is more similar to something else compared to other strains in the same population. In other words, fastGEAR infers recombination by searching for similar nucleotide segments between diverse sequence clusters. To test the significance of the inferred recombination events and identify a false-positive recombination event, fastGEAR uses a diversity test, wherein the diversity of the fragment in question is different compared to its background. Phylogenetic trees were also inferred from the multi-species *murE* – *penA* alignments using RAxML, as described above, but without recombination correction.

## Results

### Gonococcal isolates with high-level ceftriaxone and cefixime MICs carrying *penA-60* and *penA-237* alleles in the context of the global MLST-1901-associated Ng_cgc_200, Group 31

The fully reconstructed maximum likelihood (ML) phylogenetic analysis of 2,104 MLST-1901-associated core-genome group 31 gonococci global isolates (based on 17,472 non-recombinant SNP sites), sampled from 40 countries (including the US) between 1992 and 2022, was broadly partitioned into four major lineages (Figure 1), based on their monophyletic origins. These isolates belonged to 52 known MLSTs, with the most predominant MLST being 1901 (75.3%, 1,585/2,104). Lineage 1 (*n* = 212) mostly consisted of MLSTs ST7822 (*n* = 80), ST10314 (*n* = 71) and ST1901 (*n* = 18) and included isolates mostly from Europe (*n* = 143) and North America (*n* = 51). The majority of the Lineage 1 contained ESC susceptible isolates harboring non-mosaic *penA-*5 alleles (98.1%, *n* = 197). The three ST1901 gonococcal isolates with high-level MICs carrying *penA-*60 (GCWGS-10753) and *penA-*237 (F92 and H22-722) also clustered within Lineage 1. The smallest lineage, Lineage 2 (*n* = 54), consisted of mainly Asia-derived isolates (*n* = 35) harboring the mosaic *penA-10* allele, that belonged to MLSTs ST1901 (*n* = 27) and ST1579 (*n* = 25). The second largest lineage, Lineage 3 (*n* = 432), represented isolates mostly from North America (*n* = 250) and Europe (*n* = 120) and consisted primarily of MLST-1901 (*n* = 345) isolates and harboring mostly *penA-*5 (*n* = 378) among other non-mosaic alleles, which were associated with ESC susceptibility. The largest lineage, Lineage 4 (*n* = 1400), was primarily composed of isolates from Europe (*n* = 722) and North America (n = 490). Lineage 4 isolates were predominately MLST-1901 (*n* = 1187) and harbored the mosaic *penA-34*, which were associated with elevated ESC MICs (CFM; MIC ≥ 0.25 μg/ml and CRO; MIC ≥ 0.125 μg/ml) but susceptible for ESC (Supplementary Table 1). This lineage was previously well studied and was estimated to be recently expanded from a common ancestor in the 1990s [clade AB1 described in Thomas et al. (2021), clade A described in Osnes et al. (2021) and sublineage 34 described in Yahara et al. (2021)]. Additional details on the MLST-1901 Ng_cgc_200 group 31 isolates included in this study are provided in Supplementary Table 1 and Figure 1.

A focused ML phylogenetic analysis of only Lineage 1 isolates (*n* = 212) based on 4141 non-recombinant SNP sites is shown in Figure 2. The four major MLSTs in lineage 1 isolates were ST7822 (*n* = 80), ST10314 (*n* = 71), ST1901 (*n* = 19) and ST11706 (*n* = 18), and clustered into MLST-associated clades. Within each of these MLST specific clades, geographical location specific subclades were also evident throughout the phylogeny (Figure 2). Notably, the MLST-1901 clade with 19 isolates within Lineage 1 contained all three high-level ESC strains, GCWGS-10723, H22-722, and F92. This clade possessed high genetic diversity and predominantly contained isolates from North America (*n* = 8; late-2000s, mid-2010s, and 2019) and Southeast Asian countries (n = 7; early and mid-2010s) and were susceptible to ESCs as they harbored non-mosaic *penA-5* with the exception of the three high-level ESC strains. The closest genetic relative to GCWGS-10723 that carried mosaic *penA-*60 was strain 88259 (Ontario, Canada, 2008; 128 SNPs differences), which is susceptible to both cefixime and ceftriaxone. Interestingly, the estimated longer branch length of this 2019 Las Vegas, U.S. strain (GCWGS-10723) suggested that it is a highly divergent strain within Lineage 1 isolates. A genetically similar isolate is either not genome sequenced yet or not detected anywhere else. Notably, an isolate, 86394 China (2012) clustered as an outgroup to the subclade containing GCWGS-10723, 88259 and other isolates from China and the U.S. but was genetically different by 137 SNPs, compared to the Las Vegas strain, suggesting that these isolates could have an Asian ancestral origin. Strains H22-722 and F92 with mosaic *penA*-237 alleles were more genetically similar to each other (27 SNPs), as previously shown (Berçot et al., 2022), than either was to GCWGS-10723 (213 and 218 SNPs respectively). The closest genetically similar isolates to both H22-722 and F92 were isolates 86122 (China, 2012) and 116842 (New Zealand, 2019), which were on average 76 and 247 SNPs different, respectively. This MLST-1901 clade within Lineage 1 contained U.S. isolates (Hawaii, 2016) that formed a monophyletic subclade with high-level MICs for azithromycin (AZM) conferred due to the 23S rRNA 2059A→G mutation, while the Canadian isolate, 88259 that clustered with the Las Vegas isolate, possessed resistance-level MICs for AZM due to 23S rRNA 2611C→T mutations. The above-mentioned high-level AZM U.S. subclade isolates also carried non-mosaic *penA-*18 (*penA-*5 + A501T mutation; ∼99.94% sequence similarity to *penA*-5). All other MLST-specific clades were susceptible to ESC and AZM. All the lineage 1 isolates, except for one isolate (116009; USA), carried both gyrA Ser91Phe and gyrA Asp95Ala/Gly mutations that confer ciprofloxacin resistance (Supplementary Table 1 and Figure 2).

### Characterization of recombination events across *penA*-*murE* region among Lineage 1 isolates

**FIGURE 3:**
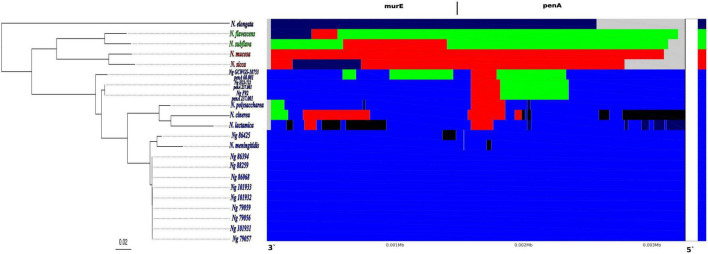
Results showing the inter-species recent recombination events detected by fastGEAR across the *Neisseria* species alignment of *murE-penA* homologous region. The phylogenetic tree on the left shows the maximum likelihood phylogenetic tree reconstructed based on the multi-*Neisseria* species *murE-penA* homologous regions. In each panel, the rows correspond to each species/isolate, columns correspond to positions in the alignment and colors show the shared genotypes of each of the *Neisseria* species included in the alignment. Each of the four lineages determined by fastGEAR, based on the multi- *Neisseria* species *murE-penA* homologous regions, are shown in 4 different colors: *N. elongata*–dark blue; *N. flavescens* and *N. subflava* -green; *N. mucosa* and *N. sicca*–red and *N. gonorrhoeae, N. polysaccharea, N. cinerea, N. lactamica and N. meningitidis*–blue. Black colored regions represent external recombination importation events that are not from any of the *Neisseria* species included in the fastGEAR analysis.

Our Gubbins-based recombination analysis on all the Lineage 1 (*n* = 212) isolates revealed the presence of multiple recombination events within the *murE-penA* region. The predicted recombination events were specifically enriched within the highly diverged MLST-1901 clade, where all three high-level ESC strains, GCWGS-10723, H22-722, and F92, clustered (Figure 3). Two unique recombination events were detected (shown as blue lines in Supplementary Figure 1): 1) 2.5 kb stretch of the genome of GCWGS-10723 covering the *dca – murE* region, but not within the *penA* region; 2) 1.2 kb stretch of the strain 116843 genome (New Zealand, 2019) covering the *murE* region. An 822 bps region at the 3′ end of the *penA* gene was also predicted to be under recombination and was shared among 11 isolates (including GCWGS-10723 but not in H22-722 or F92) through common descent (shown in red lines in Supplementary Figure 1).

FastGEAR analysis on an alignment of the *murE-penA* homologous region with other *Neisseria* species and 11 *N. gonorrhoeae* isolates (see materials and methods) revealed the presence of four lineages and five clusters. The four lineages consisted of 1) *N. elongata*, 2) *N. flavescens* and *N. subflava*, 3) *N. mucosa* and *N. sicca* and 4) all *N. gonorrhoeae* isolates*, N. polysaccharea, N. cinerea, N, lactamica and N. meningitidis.* The cluster partitions were similar to the lineage assignments, with the exception that all the three high-level ESC *N. gonorrhoeae* isolates, GCWGS-10723, H22-722, and F92, carrying *penA*-60 and *penA*-237 alleles in the lineage 4 were split into two clusters (Figure 3). Four potential interspecies recombination events were detected in the *penA-60* carrying GCWGS-10723 isolate, two within the *murE* and two within *penA* gene boundaries. The two recombination events within the *murE* gene were from the 591^st^ nucleotide to the 695^th^ nucleotide [104 bp; log (Bayes Factor (BF)) = 11.3] and from the 957^th^ to the 1451^st^ nucleotide (494 bp; log (BF) = 0.2), the latter event was only weakly supported. Both the recombination events at the *murE* gene region for the GCWGS-10723 isolate indicated a shared ancestry from *N. flavescens* and *N. subflava*, suggesting potential interspecies recombination events. Both *murE* recombination events predicted by fastGEAR overlap within the 2.5kb recombination event inferred by Gubbins. The two adjacent recombination events inferred by fastGEAR within the *penA* gene were 1) from the 925^th^ to 1464^th^ nucleotide (539 bp; log (BF) = 90.7 and the source of this recombination event was from *N. flavescens* and *N. subflava*), 2) from the 1465^th^ to 1665^th^ nucleotide (200 bp; log (BF) = 23.7 and the source of this recombination event is from *N. sicca* and *N. mucosa*). Similar to the GCWGS-10723 isolate, fastGEAR also estimated two inter-species recombination events in F92 and H22-722 isolates carrying *penA-237* alleles at approximately the same regions of the *penA* gene as identified in the Las Vegas isolate (GCWGS-10723): 1) from the 905^th^ to 1436^th^ nucleotide (531 bp; log (BF) = 102.2 and the source of this recombination event was from *N. flavescens* and *N. subflava*), 2) from the 1437^th^ to 1665^th^ nucleotide (228 bp; log (BF) = 30.9 and the source of this recombination event is from *N. sicca* and *N. mucosa*). Both of these recombination blocks were predicted exactly at the same locations in both F92 and H22-722 isolates, confirming that these two strains were highly related. Unlike the Las Vegas isolate, there were no predicted recombination events at the *murE* region for either the F92 or H22-722 isolate (Figure 3). Interestingly, *N. flavescens* is now considered to be the same species as *N. subflava* (Bennett et al., 2014; Diallo et al., 2019). Also, isolates previously classified as *Neisseria sicca* are now identified as variants of *N. mucosa* (Bennett et al., 2012). Herein onwards, both *N. flavescens* and *N. subflava* will be represented as *N. subflava*, and *N. sicca* and *N. mucosa* as *N. sicca*.

In order to validate the fastGEAR results, we generated a sequence alignment of *penA* genes from the following *N. gonorrhoeae* isolates: 88259 (*penA-5*), 79059 (*penA-18*), GCWGS-10723 (*penA-60*), F92 (*penA-237*), along with *penA* gene homologs from *N. sicca* and *N. subflava*, and visualized the nucleotide differences in the two predicted recombinant blocks towards the second half of the *penA* gene (Supplementary Figure 2 and Supplementary Table 2). Out of the 82 codon substitutions identified in the first recombinant block predicted to be inherited from *N. subflava* in both GCWGS-10723 and F92 isolates, 77 variants matched to both *N. subflava* and *N. sicca*, and 60% (46/77) of those codon variants had the nucleotide matched only to *N. subflava*, which was consistent with the source species of the inter species recombination event predicted by fastGEAR. Similarly, out of 29 codon variants identified at the second recombination block, 24 substitutions matched to *N. subflava* and *N. sicca*, and 19/24 of those codon variants had the nucleotide at the variant site, matched to only *N. sicca*, again consistent with results from fastGEAR, that *N. sicca* could be the potential donor species responsible for the second recombination event in both the *penA-60* and *penA-237* alleles. The majority of the above mutations were nonsynonymous mutations (Supplementary Figure 2 and Supplementary Table 2).

As expected, out of the five synonymous mutations between the *penA-60* and *penA-237* alleles (Berçot et al., 2022), the variant Pro328Ala in *penA-237*, which belonged to the first recombination block, appeared to be imported from *N. subflava*; while the remaining variants within the second recombination block, Ala481Pro, Ser484Thr, Ile486Thr and Thr535Ala matched to *N. sicca*, with the exception of Thr535Ala being unique in *penA-237*. There were three nonsynonymous mutations between the *penA-60* and *penA-237* alleles within the first recombination block imported from *N. subflava*, at amino acid positions 302, 329 and 330. The variant codons were similar to *N. subflava*, with the exception at amino acid position 330, where the codon was similar to both *N. subflava* and *N. sicca.* Out of the seven amino acid substitutions associated with the mosaic *penA* alleles present in *penA-60* and *penA-237* that cause ESC resistance, we were able to identify the source of the recombinant DNA for four substitutions Ile312Met (both *N. subflava* and *N. sicca*), Val316Thr (*N. subflava*) and the deletion of Asp345 (codon matched to *N. subflava*). The codons for Ala311Val, Asn513Tyr and Gly545Ser, observed in the *penA-60* and *penA-237* alleles, were different, compared to both *N. subflava* and *N. sicca*.

## Discussion

Reduced susceptibility to ESCs in *N. gonorrhoeae* has remained relatively low in the U.S. (fluctuating between 0.2 and 0.4% for CRO reduced susceptibility isolates between 2017 and 2020; declining from 1.4% in 2011 to 0.2% in 2021 for CFM reduced susceptibility isolates) (CDC, 2023). Of the U.S. isolates reported with elevated cephalosporin MICs, the majority belonged to MLST-1901 (Ng_cgc_200 group no. 31), a globally circulating lineage associated with mosaic *penA*-34 alleles (Thomas et al., 2019, 2021; Reimche et al., 2023). In 2010, a single ST1901 isolate (F89; France) possessing high-level ESC MICs was attributed to a treatment failure case, but did not spread further (Unemo et al., 2012). Furthermore, as summarized above, all recent reports of high-level ESC MICs over the last 10-years have largely been associated with the international spread of FC428 clones (Ng_cgc_400 group no. 410) harboring mosaic *penA*-60, which are genetically distinct from other gonococcal lineages. Nonetheless, there appears to be increasing reports of non-FC428 isolates possessing mosaic *penA*-60 alleles (Reimche et al., 2024), and more recently mosaic *penA-*237. This suggests that either these alleles do not impart a significant fitness cost and/or there are additional compensatory mutations present that may restore fitness (Vincent et al., 2018).

To our knowledge, the isolate GCWGS-10723, identified by SNHD, was the first *N. gonorrhoeae* isolate detected globally to date that belonged to MLST-1901 and carried a mosaic *penA-60* allele (Picker et al., 2020; Reimche et al., 2023). This isolate possesses the highest ceftriaxone MIC since GISP began monitoring antimicrobial susceptibilities in 1986. Although it belonged to the ST1901-associated core-genome group, this as well as two other MLST-1901 isolates with high-level ESC MICs (F92 and H22-722) were more closely related to the largely ESC susceptible lineage (Lineage 1). This lineage harbors non-mosaic *penA*-5 and similar derivative alleles of Asian origin, as well as the non-mosaic penA-18.001 allele detected in the U.S. (Hawaii), but are also associated with travel to Asia (Katz et al., 2017). Within Lineage 1, these isolates clustered within the MLST-1901 clade with substantial unsampled genomic diversity. This is particularly evident in the long branch lengths. The genetically closest isolates were older isolates sampled in geographically distant areas. Even though all three high-level ESC MICs MLST-1901 isolates were isolated in Europe and North America, all of the cases were directly or indirectly epidemiologically linked (via sexual partners) with travel to Asian countries (Picker et al., 2020; Berçot et al., 2022; Day et al., 2022). Interestingly, 9 out of 20 MLST-1901 clade isolates within Lineage 1 carried AMR markers suggestive of AMR resistance to more than one antibiotic, indicating the possible existence of even more concerning isolates within the MLST-1901 clade, which are most likely under sampled and possibly circulating in Asia. In addition, the concurrence of two cases of MLST-1901 carrying mosaic *penA*-237 in Europe within the same year suggests that this strain may still be circulating among sexual networks. Surprisingly, culture independent molecular testing of 257 remnant *N. gonorrhoeae* NAAT-positive specimens from all SNHD clinics collected during September to December 2019 did not identify any specimens with the mosaic *penA*-60 allele. Moreover, AST of approximately 5,500 gonococcal isolates via GISP during January-December 2019, as well as to date, did not identify any isolates with high-level ESC MICs as isolate GCWGS-10723, among MLST-1901 isolates (Picker et al., 2020). Taken together, this suggest that the MLST-1901 strain carrying the *penA*-60 allele, identified in Las Vegas (GCWGS-10723), was probably imported from Asia and was effectively detected in the U.S. Testing and adequate treatment of this patient possibly prevented further transmission among sexual networks. It is also possible that the continued dissemination of this strain did not alert public health laboratories, as this strain was still responsive to ceftriaxone treatment.

Our detailed recombination analysis of the *penA* gene suggests the possibility of two separate inter-species recombination events from *N. subflava* and *N. sicca* to the second half of the *penA* gene. This may have contributed to the generation of mosaic *penA*-60 (GCWGS-10723) and *penA-237* (H22-722 and F92) alleles within a possible *penA-5* ancestral background. Also, differences in the length of nucleotide sequences under recombination at the *penA* region in these isolates suggest two or more separate inter-species recombination events might have led to the emergence of *penA-*60 and *penA-237* alleles, rather than the latter allele being derived from the former allele. Transformation experiments have demonstrated that high-level ESC strains, such as the FC428 clones can be generated by homologous recombination of mosaic *penA* alleles from commensals, such as *N. cinerea* or *N. subflava*, into a susceptible receipt strain (Igawa et al., 2018; Kanesaka et al., 2021). While useful, these methods may not fully recapitulate all of the various pathways of homologous recombination that may arise from genetic exchange across multiple *Neisseria* species. It is also possible that high-level ESC isolates within the MLST-1901 Clade in lineage 1 acquired mosaic *penA*-60 and *penA*-237 genotypes through unique recombination events, either independent of the FC428 clones and directly from other *Neisseria* commensal species or these stains could have also originated by recombination between 2 Ng strains, one with MLST-1901 genomic background and the donor strain belonged to the FC428 clone. Confirmation of the exact path of origins of these strains is beyond the scope of this study.

The sources of origin for mosaic codon variants for *Ala311Val*, *Asn513Tyr* and *Gly545Ser* observed in mosaic *penA*-60 and *penA*-237 alleles, in these three high-level ESC isolates, were not assigned to *N. subflava* and *N. sicca* because the reference genomes analyzed for those two species, in our recombination analysis, did not carry those variants. Nevertheless, there are many reports of *N. subflava* and *N.sicca* carrying those mosaic variants, along with high-level ESC MICs, but neither whole genome data nor Sanger sequenced *penA* genes are publicly available (Kanesaka et al., 2021, 2023). Interestingly, recent cross-sectional studies assessing the resistance patterns of oropharyngeal *Neisseria* species in Italy (Gaspari et al., 2023), Vietnam (Dong et al., 2020) and Belgium (Laumen et al., 2021) identified *N. subflava* and *N. flavescens* as the most common pharyngeal non-pathogenic *Neisseria* species. Even though substantial effort is spent on identifying resistance mechanisms circulating within the gonococcal populations, these surveys often overlook a known source of resistance for gonococci−i.e. the commensal *Neisseria* species. The diversity of AMR alleles in commensal population is currently unknown, presenting an issue for the complete genotypic-based resistance prediction for any of the *Neisseria* species, including pathogens. As reviewed and suggested in Goytia and Wadsworth (2022), improved characterization of the *Neisseria* resistome, and subsequent surveillance of commensal populations could serve as a “canary in the coal mine” approach to identifying new or common resistance mechanisms that may emerge from our microbial reservoirs (Frost et al., 2023; Unitt et al., 2024).

Furthermore, as observed in the FC428 clones, it is possible that there may be future cases involving ST1901-associated genome group as many already possess *gyrA* mutations shown experimentally to compensate for fitness costs associated with mosaic *penA* alleles (Kanesaka et al., 2021). In fact, this phenomenon is already happening because almost all of the non-FC428 clone lineages carrying *penA*-60 alleles, such as MLSTs-8123, 12039, 16406 and 8143, also carried *gyrA* mutations associated with ciprofloxacin resistance (Reimche et al., 2024; Pleininger et al., 2022; Maubaret et al., 2023).

The recent emergence of gonococcal isolates with high level ESC in historically susceptible lineages is a major concern, especially considering that ceftriaxone is the only remaining first-line treatment for gonorrhea. Our study underscores the necessity of ongoing surveillance of both gonococcal and commensal *Neisseria* populations, public health action, and the need for point-of-care testing to monitor trends in antimicrobial resistance. Notably, it is critically important to enhance AMR surveillance in countries that may be historically under sampled for gonococcal AMR surveillance.

## *Neisseria gonorrhoeae* working group

**Division of STD Prevention, Centers for Disease Control and Prevention, Atlanta, GA, USA**

Kim Gernert, Matthew W. Schmerer and Cau D. Pham

**Washington State Department of Health, Washington State Regional Lab, Shoreline, WA, USA**

Mike Tran, Sopheay Hun, Soge O Olusegun, Chi Hua, Brian Hiatt and Kirstin Veliz

**Tennessee Department of Health, Tennessee Regional Lab Nashville, Nashville, TN, USA**

Lindsay Jolly and Maya Spann

**Texas Department of State Health Services, Texas Regional Lab Austin, Austin, TX, USA**

Tamara Baldwin, Chun Wang, Maliha Rahman and Bonnie Oh

**Maryland Department of Health, Maryland Regional Lab, Baltimore, MD, USA**

David Torpey, Teisha Leak, Rebecca Abelman, Eric Keller, Terence Moore and Jillian Loomis.

**Utah Department of Health and Human Services, Utah Public Health Laboratory, Salt Lake City, UT, USA**

Lindsay Neff, Ravyn Casey, Jenni Wagner, Erin Young, and Kelly F. Oakeson

## Data Availability

The datasets presented in this study can be found in online repositories. The names of the repository/repositories and accession number(s) can be found in the article/Supplementary material.

## Footnotes

1
https://github.com/marbl/Mash/releases/tag/v2.2

2
https://sourceforge.net/projects/bbmap/

3
https://github.com/sanger-pathogens/ariba

4
https://github.com/HadrienG/InSilicoSeq

5
https://github.com/tseemann/mlst

6
https://github.com/MDU-PHL/ngmaster

7
https://github.com/leosanbu/pyngSTar

8
https://pubmlst.org/neisseria

9
https://github.com/tseemann/snippy

10
https://github.com/stamatak/standard-RAxML

11
https://github.com/tseemann/snp-dists

12
https://github.com/rcedgar/muscle

## Supplementary material

Supplementary Figure 1 Visualization of homologous recombination events identified by Gubbins in and around the *murE-penA* region of 213 MLST-1901-associated core-genome group 31 lineage 1 gonococci global isolates. The phylogenetic tree on the left is the maximum likelihood tree inferred on the whole genome alignment by Gubbins and RAxML. The colored strips show the metadata for the isolates in the phylogenetic tree. Horizontal tracks showing genomic locations containing putative recombination events identified using Gubbins. Recombination events colored in red were found in multiple isolates at the same genomic coordinate, while those shown in blue were found in a single genome. Note that unique recombination events found in different genomes and those found at distinct locations in the same genome are both colored in blue. Arrows to show genes in the forward and reverse strand of the annotated reference genome. Graph showing the number of independent recombinations per isolate in the specified region on the alignment.

Supplementary Figure 2 Visualization of the alignment of the homologous *penA* gene region in *N. subflava*, *N. sicca, penA-5.001, penA-18.001, penA-60* and *penA-237*alleles. The nucleotide and amino acid (codons) are shown. The alignment was generated using the Geneious aligner with the default parameters. The top row shows the consensus sequences (both amino acid and nucleotide sequence) at each aligned position. The second row shows identity (green color represents 100% identity) across the homologous *penA* gene region for the 2 commensal species and 4 different Ng *penA* allele gene sequences. The mutations with in each of the sequences both at amino acid and nucleotide levels are shown in different colors, along with the alignment positions. Important variants identified towards the second half of the *penA* gene across all the 6 sequences and their potential inherited is shown in Supplementary Table 2.

Supplementary Table 1 Isolate information with all associated metadata and antimicrobial (AMR) variants associated with all isolates used in this study.

Supplementary Table 2 Variants identified towards the second half of the *penA* gene, across the alignment with the *penA*-homologous regions among *N. subflava*, *N. sicca, penA-5.001, penA-18.001, penA-60*and *penA-237* alleles and potential inheritance of each variant. This table was meant to validate the inter-species recombination events inferred through fastGEAR analysis.
