# Supplementary figures and images for: Emergence and evolution of mosaic penA-60 and penA-237 alleles in a Neisseria gonorrhoeae core genogroup that was historically susceptible to extended spectrum cephalosporins
Source: Front Microbiol. 2024 Oct 1;15:1401303. doi: 10.3389/fmicb.2024.1401303 (PMC11473337; doi:10.3389/fmicb.2024.1401303)

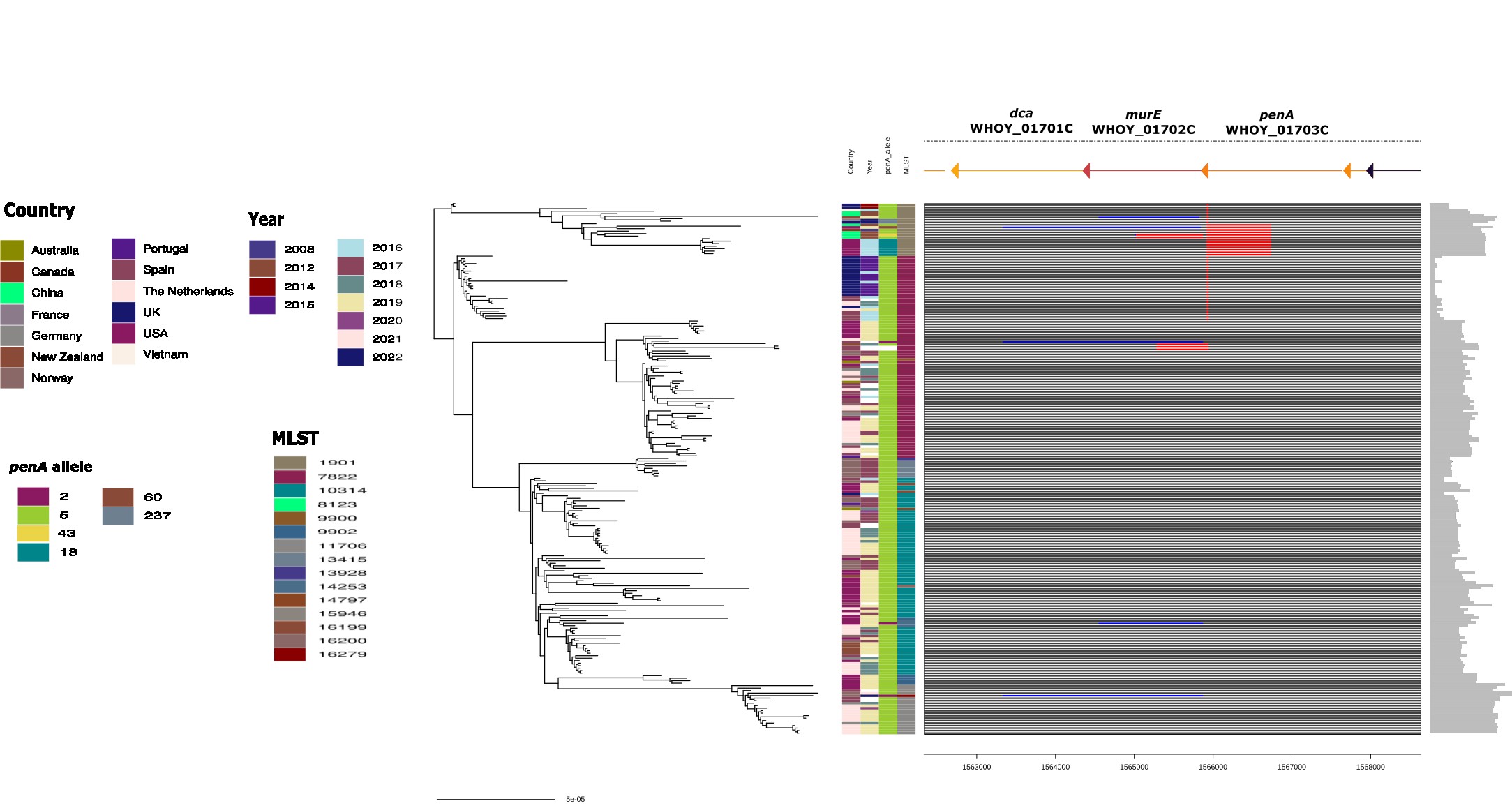

Supplement: Supplementary Figure 1 — Visualization of homologous recombination events identified by Gubbins in and around the murE-penA region of 213 MLST-1901-associated core-genome group 31 lineage 1 gonococci global isolates. The phylogenetic tree on the left is the maximum likelihood tree inferred on the whole genome alignment by Gubbins and RAxML. The colored strips show the metadata for the isolates in the phylogenetic tree. Horizontal tracks showing genomic locations containing putative recombination events identified using Gubbins. Recombination events colored in red were found in multiple isolates at the same genomic coordinate, while those shown in blue were found in a single genome. Note that unique recombination events found in different genomes and those found at distinct locations in the same genome are both colored in blue. Arrows to show genes in the forward and reverse strand of the annotated reference genome. Graph showing the number of independent recombinations per isolate in the specified region on the alignment. [file Image_1.JPEG]

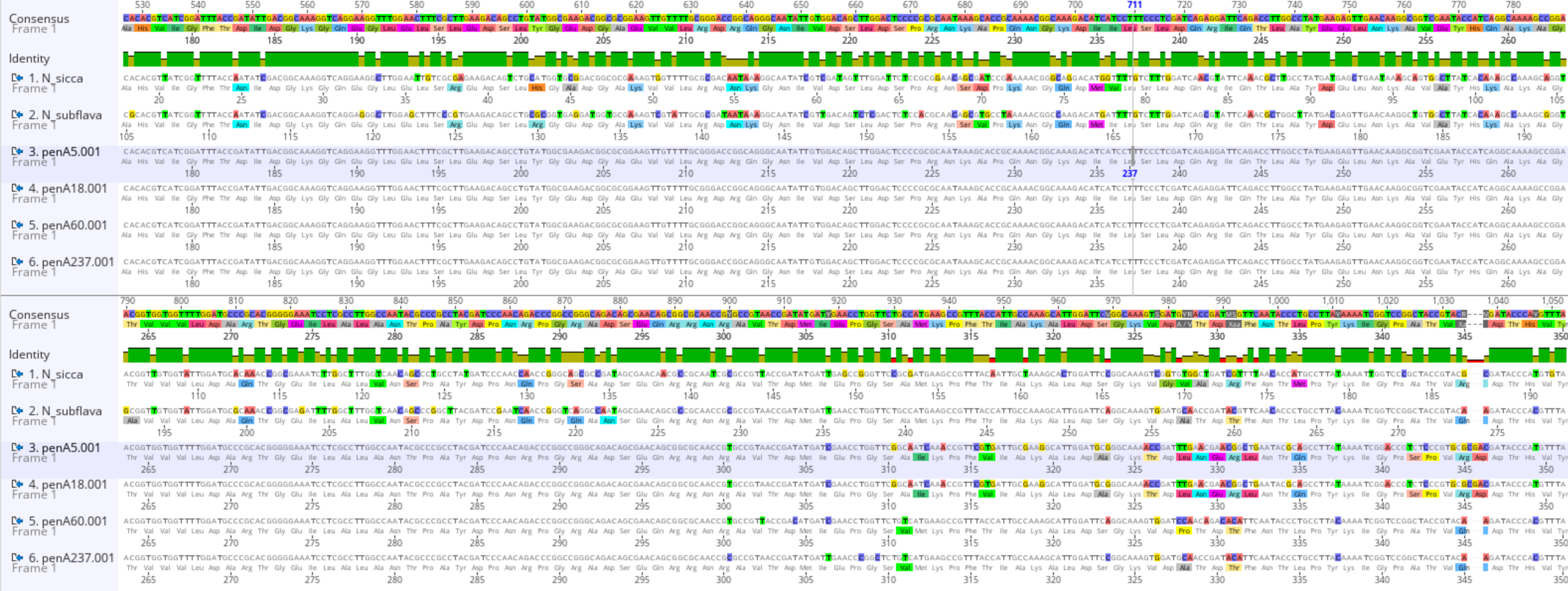

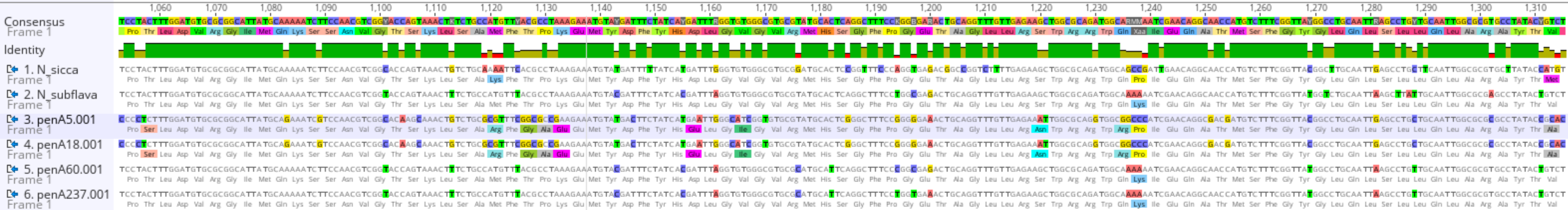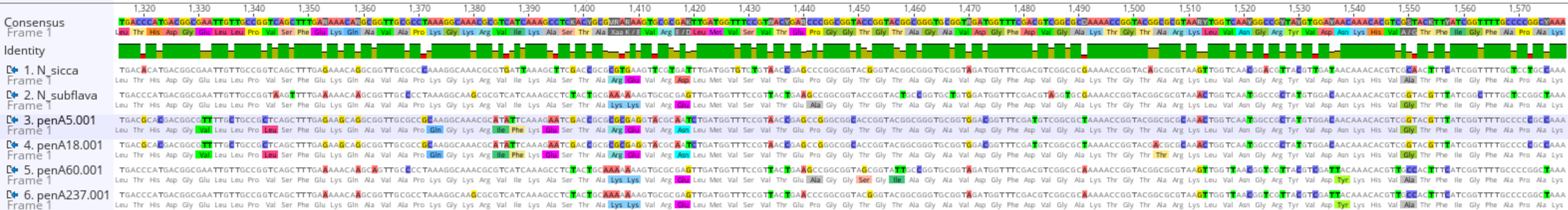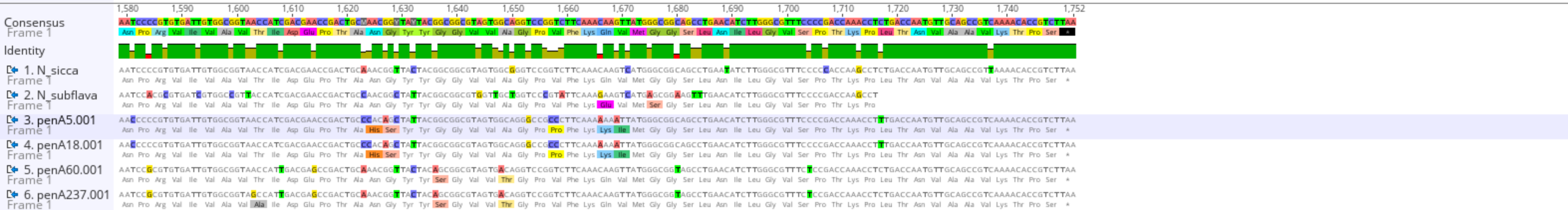

Supplement: Supplementary Figure 2 — Visualization of the alignment of the homologous penA gene region in N. subflava, N. sicca, penA-5.001, penA-18.001, penA-60 and penA-237alleles. The nucleotide and amino acid (codons) are shown. The alignment was generated using the Geneious aligner with the default parameters. The top row shows the consensus sequences (both amino acid and nucleotide sequence) at each aligned position. The second row shows identity (green color represents 100% identity) across the homologous penA gene region for the 2 commensal species and 4 different Ng penA allele gene sequences. The mutations with in each of the sequences both at amino acid and nucleotide levels are shown in different colors, along with the alignment positions. Important variants identified towards the second half of the penA gene across all the 6 sequences and their potential inherited is shown in Supplementary Table 2. [file Image_2.PDF]
